# Effect of Traditional Chinese Medicine Therapy on the Trend in CD4^+^ T-Cell Counts among Patients with HIV/AIDS Treated with Antiretroviral Therapy: A Retrospective Cohort Study

**DOI:** 10.1155/2021/5576612

**Published:** 2021-07-15

**Authors:** Dongli Wang, Suna Ma, Yanmin Ma, Huijun Guo, Pengyu Li, Chunling Yang, Qianlei Xu, Zhibin Liu, Yantao Jin

**Affiliations:** ^1^The First Clinical Medical School, Henan University of Chinese Medicine, Zhengzhou 45000, China; ^2^Center for AIDS/STD Control and Prevention, Center for Disease Control and Prevention of Henan Province, Zhengzhou 45000, China; ^3^Department of Acquired Immune Deficiency Syndrome Treatment and Research Center, The First Affiliated Hospital of Henan University of Chinese Medicine, Zhengzhou 45000, China

## Abstract

This retrospective cohort study was conducted to explore the effect of traditional Chinese medicine (TCM) therapy on the long-term trends in CD4^+^ T-cell count among patients with human immunodeficiency virus/acquired immunodeficiency syndrome (HIV/AIDS) who were treated with combined antiretroviral therapy (cART) over a 14-year period. A total of 721 individuals were treated with cART alone (cART group), and 307 individuals were treated with both cART and TCM (TCM + cART group). Among all enrolled patients with HIV/AIDS, 99.5% were farmers, 71.1% had more than 6 years of education, and 96.8% were infected with HIV via a paid blood donation. For those patients with HIV/AIDS who had a baseline CD4^+^ T-cell count of <350 cells/mL, the CD4^+^ T-cell count tended to increase to approximately 350 cells/mL more rapidly in the TCM + cART group than in the cART group, but when the baseline CD4^+^ T-cell count was ≥350 cells/mL, there was no difference between the cART and TCM + cART groups. For other patients with HIV/AIDS who had a baseline CD4^+^ T-cell count of 350–500 cells/mL, the CD4^+^ T-cell counts tended to increase slightly, but there was no difference between the two groups. For patients with HIV/AIDS who had a baseline CD4^+^ T-cell count of ≥500 cells/mL, the CD4^+^ T-cell counts tended to be maintained at a particular level, with no difference between the two groups. The results show that the effect of TCM on the CD4^+^ T-cell counts of patients with HIV/AIDS is related to the CD4^+^ T-cell level at the time of initial treatment. TCM can increase the CD4^+^ T-cell count among patients with HIV/AIDS who have a baseline CD4^+^ T-cell count of <350 cells/mL. Sex and age have a slight influence on the therapeutic effect of TCM.

## 1. Introduction

Acquired immunodeficiency syndrome (AIDS) is a highly contagious disease caused by infection with human immunodeficiency virus (HIV) [1]. CD4^+^ T-lymphocytes are the main cellular target of HIV. Because these cells form an important part of the human immune system, damage to them can reduce immune function and lead to opportunistic infections and malignant tumors [2]. CD4^+^ T-cell counts play an important role in treatment decisions and clinical management for patients with HIV/AIDS, especially for patients in places with limited resources and those who present late for care [3] and in treatment monitoring where viral load monitoring is restricted [4].

Combined antiretroviral therapy (cART) is a standard medical treatment for HIV infection and AIDS symptom control [5]. In response to the HIV/AIDS epidemic in China, the Chinese government has gradually promoted free cART therapy among patients with HIV/AIDS. Through constant effort, the HIV epidemic in China has been controlled remarkably, and the immunity of patients with HIV/AIDS has been greatly enhanced [6]. However, cART can only inhibit, but not eradicate, the disease; consequently, patients with HIV/AIDS must take medication for a long time, even for the rest of their lives [7]. China is still one of the largest AIDS-epidemic countries in the world, and the number of new cases detected continues to increase each year [6]. Traditional Chinese Medicine (TCM) has been used to treat infectious diseases for thousands of years [8]. Previous studies have demonstrated that TCM can increase CD4^+^ T-cell counts and improve the immunological function of patients with HIV/AIDS [9], but most of these studies were small, with low sample sizes and short observation times. There is little available data on the long-term trends in CD4^+^ T-cell counts among patients with HIV/AIDS receiving TCM therapy.

In the mid-1990s, some farmers in poor rural communities in China sold their plasma to unscrupulous buyers under unsanitary conditions, resulting in the infection with HIV of approximately 69,000 individuals. Henan Province and the adjacent areas of surrounding provinces are the central regions of the HIV epidemic area [10]. In response, the Chinese government established the National Free Antiretroviral Treatment Program (NFATP) and Nation Free TCM HIV/AIDS Treatment program (NTCMTP) to provide free cART and TCM to patients with HIV/AIDS, and they used standard medical registers to record patient information [11]. In the present study, we used these medical data to analyze the annual trends in CD4^+^ T-cell counts to evaluate the curative effect of TCM on the treatment of patients with HIV/AIDS who were also receiving cART therapy.

## 2. Methods

### 2.1. Study Design, Setting, and Population

This 14-year retrospective cohort study was conducted in Henan Province, China, an area where patients with HIV/AIDS received cART via the NFATP. Most patients with HIV/AIDS in 2004 were infected through the paid blood supply or illegal blood plasma collection during the 1990s [10]. Henan was one of the earliest areas to begin the distribution of free cART via the NFATP and free TCM therapy via the NTCMTP. The subset of study patients who participated in the NTCMTP did so voluntarily and signed the informed consent. All patients who participated in the NTCMTP of Henan Province were given the free patented TCM preparation “yi ai kang capsule” (containing substances such as ginseng, huangqi, chaobaishu, Tuckahoe, Chinese angelica, chuanxiong, baishao, and Scutellariae), prescribed as five capsules, taken three times per day, and they established medical records to record their AIDS-related information monthly [12]. The NFATP and NTCMTP were conducted by different departments with mutual noninterference. Information about the individuals in this study was collected from the two standard medical record registers from the NFATP and NTCMTP.

This cohort study was conducted on records collected from April 2004 to April 2019. All individuals had been identified by western blot as being HIV-infected and received cART during the period from April 2004 to April 2005, were older than 18 years but younger than 65 years, and had their CD4^+^ T-cell count recorded at least twice over the 14-year follow-up period. Individuals without a recorded baseline CD4^+^ T-cell count were excluded from this study. The patients were divided into the TCM + cART and cART groups on the basis of their participation in the NTCMTP, i.e., all individuals were enrolled in the NFATP, and individuals in the TCM + cART group were enrolled in the NTCMTP in October 2004.

### 2.2. Data Collection and Variables

Demographic and medical information for the study subjects, including their age, sex, marital status, race, education, occupation, route of infection, year in which their HIV diagnosis was confirmed, year in which they began ART, consumption of TCM, year of death, and CD4^+^ T-cell counts at baseline and annually thereafter, were collected. Data on the consumption of TCM was obtained from the register of the NTCMTP, the CD4^+^ T-cell count records were obtained from the registers of the NFATP and NTCMTP, and data on all other variables were obtained from the register of the NFATP.

### 2.3. Data Analysis

The mean CD4^+^ T-cell count recorded within 6 months of the annual anniversary date of cohort commencement was used as the CD4^+^ T-cell count value in the study analysis. The annual CD4^+^ T-cell count trends for the TCM + cART and cART groups were determined and stratified by the patient baseline CD4^+^ T-cell count (<200, 200–350, 350–500, and >500 cells/mL). The subgroup annual CD4^+^ T-cell count trends, stratified by sex (male versus female) or age (<45 versus >45 years old), were analyzed among patients with a baseline CD4^+^ T-cell count of <200 cells/mL or 200–350 cells/mL. The figures displaying the annual CD4^+^ T-cell count trends were drafted by using the ggplot package of R 3.6.1.

Categorical variables are reported as whole numbers with proportions, and continuous variables are reported as the mean with standard deviation (SD), unless indicated otherwise. The demographic characteristics and annual CD4^+^ T-cell count of patients in the TCM + cART group were compared with those of patients in the cART group by conducting a chi-squared test or Student's *t*-test.

All statistical analyses were performed by using R 3.6.1, and two-sided *P*

values of less than 0.05 were considered to indicate statistical significance.

### 2.4. Ethical Considerations

This study was approved by the Institutional Review Board of the First Affiliated Hospital of Henan University of Chinese Medicine (2019HL-068). Individual informed consent was not achieved because this analysis was conducted on existing data collected during the course of routine treatment, and the data are reported in aggregate without the use of individual identifying information.

## 3. Results

### 3.1. Study Population Characteristics

A total of 1,028 patients with HIV/AIDS whose records were included in the relevant registers met our criteria, and their records were extracted for analysis. Among them, the 721 individuals were included in the cART group, and the 307 individuals were included in the TCM + cART group. The mean age of all included patients with HIV/AIDS was 40.3 (SD, 8.09) years; 55.7% of these patients were younger than 40 years, 49.5% were male, 79.3% had been married, and 71.1% had more than 6 years of education. Most (99.5%) of these patients were farmers, and almost all (96.8%) had been infected with HIV via blood transmission. The cumulative mortality rate of all study patients was 27.7%. The numbers of patients with baseline CD4^+^ T-cell counts of <200 cells/mL, 200–350 cells/mL, 350–500 cells/mL, and >500 cells/mL were 256, 318, 250, and 204, respectively. Except for the baseline CD4^+^ T-cell count, there was no difference in the other assessed variables between the two groups. The demographic and baseline clinical characteristics of the study subjects are shown in Table 1.

### 3.2. Annual CD4^+^ T-Cell Count on Anniversary Date

The CD4^+^ T-cell count of the patients with HIV/AIDS increased from a baseline mean of 352 (SD, 209) to a mean of 476 (SD, 204) over the fourteen years of follow-up. The CD4^+^ T-cell counts of the TCM + cART and cART groups were significantly different from one another during the first five years of treatment. The annual number of HIV/AIDS cases and mean anniversary CD4^+^ T-cell count are shown in Table 2.

### 3.3. The Annual CD4^+^ T-Cell Count Trends in the TCM + cART and cART Groups, as Stratified by Baseline CD4^+^ T-Cell Count

**Figure 1:**
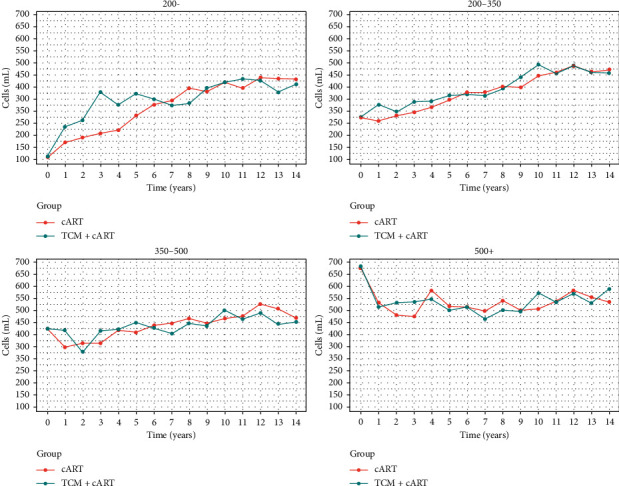
Annual CD4^+^ T-cell count trends in the TCM + cART and cART groups, as stratified by baseline CD4^+^ T-cell count (<200, 200–350, 350–500, and >500 cells/mL).

For patients with HIV/AIDS who had a baseline CD4^+^ T-cell count of <200 cells/mL or 200–350 cells/mL, the CD4^+^ T-cell count increased significantly during the early stage of treatment, with the increase occurring faster in the TCM + cART group than in the cART group, and when the CD4^+^ T-cell count reached approximately 350 cells/mL, the difference between the two groups disappeared and the rate of increase slowed down. For patients with HIV/AIDS who had a baseline CD4^+^ T-cell count of 350–500 cells/mL, the CD4^+^ T-cell count increased slightly, with no significant difference between the two treatment groups. For patients with HIV/AIDS who had a baseline CD4^+^ T-cell count of >500 cells/mL, after a brief decline, the CD4^+^ T-cell count was maintained at the same level, with no significant difference between the two treatment groups. The details of these changes are shown in Figure 1. The data of Figure 1 are shown in S1.

### 3.4. The Annual CD4^+^ T-Cell Count Trends in the TCM + cART and cART Groups, as Stratified by Patient Sex

**Figure 2:**
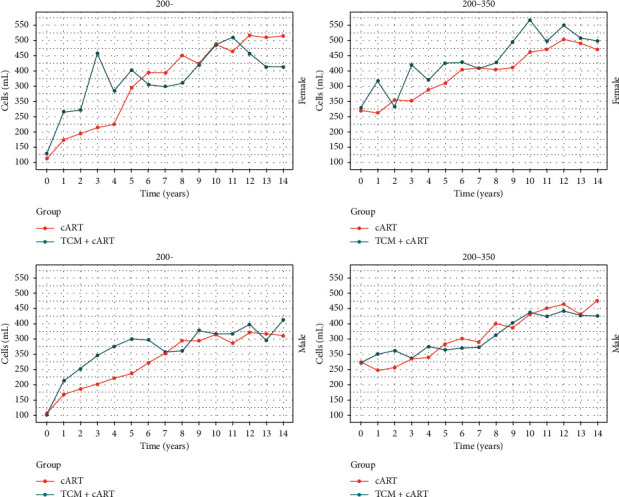
Annual CD4^+^ T-cell count trends in the TCM + cART and cART groups as stratified by both baseline CD4^+^ T-cell count (<200 and 200–350 cells/mL) and sex (male and female patients).

The CD4^+^ T-cell count of patients with HIV/AIDS increased to approximately 350 cells/mL more rapidly in the TCM + cART group than in the cART group; after the CD4^+^ T-cell count reached this level, the rate of increase slowed down, and there was no difference between the two treatment groups. Female patients reached a CD4^+^ T-cell count of approximately 350 cells/mL at a faster rate and had a higher final CD4^+^ T-cell count compared with male patients. The annual CD4^+^ T-cell count trends as stratified by sex are shown in Figure 2. The data of Figure 2 are shown in S2.

### 3.5. The Annual CD4^+^ T-Cell Count Trends in the TCM + cART and cART Groups, as Stratified by Age

**Figure 3:**
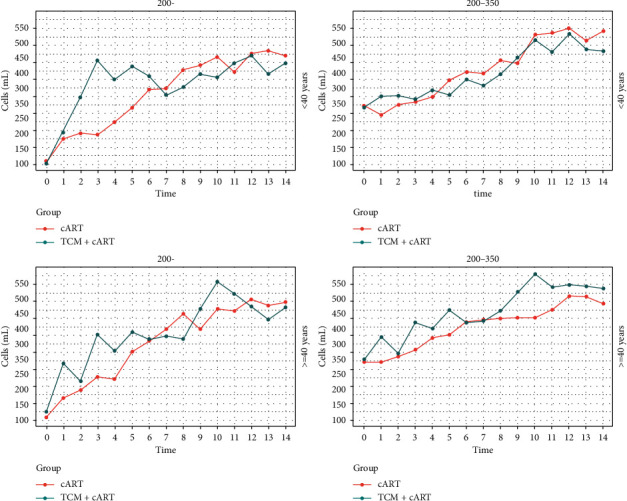
Annual CD4^+^ T-cell count trends in the TCM + cART and cART groups as stratified by both baseline CD4^+^ T-cell count (<200 and 200–350 cells/mL) and patient age (<45 and >45 years old).

The CD4^+^ T-cell count of patients with HIV/AIDS increased to approximately 350 cells/mL more rapidly in the TCM + cART group than in the cART group. For patients with HIV/AIDS who had a baseline CD4^+^ T-cell count of 200–350 cells/mL, older patients reached a CD4^+^ T-cell count of approximately 350 cells/mL at a faster rate compared with younger patients in the TCM + cART group. The annual CD4^+^ T-cell count trends as stratified by age are shown in Figure 3. The data of Figure 3 are shown in S3.

## 4. Discussion

Previous works have demonstrated that cART can effectively suppress HIV viral replication [13], can control viral load [14], and has great significance for increasing the CD4^+^ T-cell count and recovering CD4^+^ T-lymphocyte function [15, 16]. However, the benefits of cART are affected by many factors. The CD4^+^ T-cell count at the time of initial cART treatment of patients with HIV/AIDS affects the CD4^+^ T-cell count recovery and the function of the CD4^+^ T cells after receiving cART [17]. Comparison between the recovered CD4^+^ T-cell count post-cART treatment, and the baseline CD4^+^ T-cell count is necessary for monitoring the dynamic CD4^+^ T-cell count and optimizing the level of immune function in patients with HIV/AIDS [3].

In China, TCM is a mainstream type of medicine. Unlike cART, TCM works on the overall immune function of patients; this approach is important because better immune function decreases the risk of opportunistic infection and tumor formation [18]. Many studies have reported their findings regarding the influence of TCM on the immune function of patients with AIDS, but their results are inconsistent [19–21]. The influence of TCM on the CD4^+^ T-cell count of patients with HIV/AIDS might be significantly related to the CD4^+^ T-cell count at the time of initial treatment. In our study, for patients with HIV/AIDS who had a baseline CD4^+^ T-cell count of <350 cells/mL, and especially for patients with HIV/AIDS who had a baseline CD4^+^ T-cell count of <200 cells/mL, treatment with TCM plus cART increased the CD4^+^ T-cell count to 350 cells/mL more rapidly compared with treatment with cART alone; however, the difference between treatment groups disappeared once the CD4^+^ T-cell count reached approximately 350 cells/mL. For patients with HIV/AIDS who had a baseline CD4^+^ T-cell count of ≥350 cells/mL, the CD4^+^ T-cell count slightly increased or stabilized following treatment initiation, and there was no significant difference between the cART and TCM + cART groups. These results are consistent with those of an 84-month study among 110 patients with HIV/AIDS in 19 provinces and cities of China.^18^ The lack of obvious increase in CD4^+^ T-cell count after this count reached approximately 350 cells/mL maybe because CD4^+^ T-cell counts reach a plateau after treatment, as reported by some studies [22–24]. Additionally, previous works have shown that the longer a patient's CD4^+^ T-cell count remained stable before reaching 350 cells/mL, the higher the mortality rate [25]. Thus, we suggest initiating TCM therapy as early as possible among patients with HIV/AIDS to decrease mortality.

We also observed the interesting phenomenon that only patients with HIV/AIDS who had a baseline CD4^+^ T-cell count of >500 cells/mL were able to keep a CD4^+^ T-cell count of >500 cells/mL over most of the time after initiating therapy. Another study also found that patients with HIV/AIDS who had a CD4^+^ T-cell count of >500 cells/mL at the time of treatment initiation kept their CD4^+^ T-cell count at >500 cells/mL over the seven years after initiating treatment [26]. Thus, the initiation of antiretroviral therapy at a CD4^+^ T-cell count of >500 cells/mm³ might result in a higher subsequent CD4^+^ T-cell count. Additionally, previous studies have shown that those starting cART therapy with a baseline CD4^+^ T-cell count of >500 cells/mm³ could reach higher CD4^+^ T-cell counts and rates of HIV RNA suppression [27] and also had lower mortality [28].

Sex and age have been identified as factors that affect the CD4^+^ T-cell count after initiating cART [29]. In this study, we found that patient sex and age have a slight effect on the therapeutic effect. Compared with male patients, female patients reach a CD4^+^ T-cell count of approximately 350 cells/mL more quickly, and their CD4^+^ T-cell count can be maintained at a high level. This finding is consistent with other studies reporting that female patients have a better CD4^+^ T-cell count recovery [30, 31]. This difference may be related to patient physiological characteristics, treatment methods [23], and treatment compliance; however, more research is needed to address this question. Patients of different ages have different physiological functions, understanding of AIDS, and treatment compliance; consequently, their recovery of immune function also differs [29]. We found that in individuals aged over 40 years in the TCM + cART group, it took even less time for their CD4^+^ T-cell count to reach 350 cells/mL; the reason for this may be related to better treatment compliance in patients over 40 years of age.

There are some deficiencies in our research. First, in the early stage of treatment, the medical registers were not perfect and missing data, especially for the CD4^+^ T-cell count, was common. Thus, the CD4^+^ T-cell count records of patients with HIV/AIDS were often not available for every year. Second, because of the lack of annual CD4^+^ T-cell count, it was not possible to assess the difference in annual CD4^+^ T-cell count trends between the TCM + cART and cART groups by a hypothesis test. Third, our study is a retrospective cohort study; as such, some baseline variables related to the research results were not fully recorded, and there may be a selection bias and information bias that could affect the results of the study.

## 5. Conclusion

The influence of TCM on the CD4^+^ T-cell count of patients with HIV/AIDS is related to the CD4^+^ T-cell level at the time of initial treatment. TCM could increase the CD4^+^ T-cell count among patients with HIV/AIDS who had a baseline CD4^+^ T-cell count of <350 cells/mL. Sex and age had a slight influence on the therapeutic effect of TCM. We recommend initiating TCM therapy as early as possible in patients with HIV/AIDS who have a CD4^+^ T-cell count of <350 cells/mL.

## Figures and Tables

**Table 1:** Baseline demographic characteristics of the patients with HIV/AIDS in the study cohort.

Variables	All (*N* = 1028)	TCM + cART (*N* = 307)	cART (*N* = 721)	*P*
Gender				0.634
Male	509 (49.5%)	151 (49.2%)	368 (51.0%)	
Female	519 (50.5%)	156(50.8%)	353 (49.0%)	

Age (years) (SD)	40.3 (8.09)	40.8 (8.39)	40.0 (7.96)	0.196
<40 years	573 (55.7%)	167 (54.4%)	406 (56.3%)	0.619
≥40 years	455 (44.3%)	140(45.6%)	315 (43.7%)	

Marital status				0.751
Married	815 (79.3%)	241(78.5%)	574 (79.6%)	
Single/widow	213 (20.7%)	66 (21.5%)	147 (20.4%)	

Occupation				1.000
Farmer	1023 (99.5%)	306 (99.7%)	717 (99.4%)	
Others	5 (0.49%)	1 (0.33%)	4 (0.55%)	

Education level				0.383
<6 years	297 (28.9%)	95 (30.9%)	202 (28.0%)	
≥6 years	731 (71.1%)	212 (69.1%)	519 (72.0%)	

Route of infection				0.605
Plasma	995 (96.8%)	298 (97.1%)	697 (96.7%)	
Sex	16 (1.56%)	3 (0.98%)	13 (1.80%)	
Others	17 (1.65%)	6 (1.95%)	11 (1.53%)	

CD4^+^ T-cell count (cells/mL)			<0.001	
<200	256 (24.9%)	50 (16.3%)	206 (28.6%)	
200–350	318 (30.9%)	94 (30.6%)	224 (31.1%)	
350–500	250 (24.3%)	84 (27.4%)	166 (23.0%)	
>500	204 (19.8%)	79 (25.7%)	125 (17.3%)	

Death				0.247
No	743 (72.3%)	230 (74.9%)	513 (71.2%)	
Yes	285 (27.7%)	77 (25.1%)	208 (28.8%)	

**Table 2:** Annual anniversary CD4^+^ T-cell count (cells/mL) in the study cohort.

	All		TCM + cART		cART		
	*n*	Mean (SD)	*n*	Mean (SD)	*n*	Mean (SD)	*P* value
Base	1028	352 (209)	307	394 (210)	721	333 (206)	<0.001
y1	412	328 (214)	160	381 (230)	252	295 (195)	<0.001
y2	750	333 (196)	262	357 (214)	488	321 (185)	0.021
y3	734	352 (206)	249	414 (235)	485	320 (182)	<0.001
y4	674	387 (220)	250	416 (222)	424	370 (218)	0.009
y5	869	391 (196)	271	425 (191)	598	376 (197)	0.001
y6	851	409 (203)	269	418 (198)	582	405 (205)	0.397
y7	841	405 (198)	266	394 (184)	575	410 (204)	0.275
y8	837	438 (202)	263	426 (196)	574	443 (204)	0.232
y9	826	432 (193)	256	445 (195)	570	426 (192)	0.188
y10	809	471 (207)	250	503 (205)	559	456 (207)	0.003
y11	777	466 (206)	243	473 (200)	534	463 (209)	0.566
y12	758	503 (221)	236	499 (219)	522	504 (222)	0.749
y13	724	477 (205)	231	460 (203)	493	485 (205)	0.123
y14	739	476 (204)	233	481 (206)	506	474 (203)	0.661

## Data Availability

The data used to support the findings of this study are available from the corresponding author upon request.

## Supplementary Materials

Supplementary Materials S1: the annual CD4^+^ T-cell count stratified on baseline CD4^+^ T-cell count. S2: the annual CD4^+^ T-cell count stratified on gender. S3: the annual CD4^+^ T-cell count stratified on age.
