# Supplementary material for: Effect of Traditional Chinese Medicine Therapy on the Trend in CD4+ T-Cell Counts among Patients with HIV/AIDS Treated with Antiretroviral Therapy: A Retrospective Cohort Study
Source: Evid Based Complement Alternat Med. 2021 Jul 15;2021:5576612. doi: 10.1155/2021/5576612 (PMC8302365; doi:10.1155/2021/5576612)
Supplement: Supplementary Materials — S1: the annual CD4+ T-cell count stratified on baseline CD4+ T-cell count. S2: the annual CD4+ T-cell count stratified on gender. S3: the annual CD4+ T-cell count stratified on age. [file 5576612.f1.zip › 5576612.f1/S1. The annual CD4+T cell count stratified on baseline CD4+T cell count.pdf]

group	cd4cat	time	n	mean	sd	se	
1	cART	200-	cd4b	206	120.6067961	58.22966097	4.057052721
2	cART	200-	y1	78	171.1923077	153.0218818	17.32631625
3	cART	200-	y2	118	189.5805085	130.7971718	12.04085565
4	cART	200-	y3	126	206.0304233	155.1603119	13.82277922
5	cART	200-	y4	101	221.9405941	145.7757993	14.50523417
6	cART	200-	y5	150	281.6835556	175.7587996	14.35064589
7	cART	200-	y6	143	326.1369464	176.9508865	14.7973765
8	cART	200-	y7	134	344.5901741	198.2271688	17.12421922
9	cART	200-	y8	132	394.4054293	207.6654469	18.07495711
10	cART	200-	y9	132	381.0574495	202.8240446	17.65356713
11	cART	200-	y10	130	420.0897436	208.3462571	18.27317556
12	cART	200-	y11	121	393.7612397	206.1070841	18.73700765
13	cART	200-	y12	119	439.047619	211.7997459	19.41565087
14	cART	200-	y13	115	435.0666667	205.66996	19.17882266
15	cART	200-	y14	114	432.2236842	206.381408	19.32938985
16	cART	200-350	cd4b	224	272.484375	45.71042091	3.054155965
17	cART	200-350	y1	70	256.9785714	119.1060737	14.23589868
18	cART	200-350	y2	144	280.5775463	139.8123144	11.6510262
19	cART	200-350	y3	149	294.0324385	136.9603191	11.22022653
20	cART	200-350	y4	131	316.2633588	156.1286725	13.64102545
21	cART	200-350	y5	193	348.0007032	170.2462942	12.25459641
22	cART	200-350	y6	190	379.2020175	185.2689727	13.44082396
23	cART	200-350	y7	179	376.9771881	180.7891942	13.51281881
24	cART	200-350	y8	180	401.2083333	181.0715421	13.4962759
25	cART	200-350	y9	178	398.9128277	183.477	13.75218257
26	cART	200-350	y10	177	446.7448211	203.1740279	15.27149511
27	cART	200-350	y11	164	459.516001	198.7236257	15.5177081
28	cART	200-350	y12	163	486.4652352	219.2811226	17.17542307
29	cART	200-350	y13	153	463.375817	192.6349953	15.57361633
30	cART	200-350	y14	157	471.39862	208.9989611	16.67993297
31	cART	350-500	cd4b	166	422.8192771	45.21266773	3.509183583
32	cART	350-500	y1	65	344.1846154	133.0839599	16.50703365
33	cART	350-500	y2	123	361.5596206	141.9655656	12.80060397
34	cART	350-500	y3	122	360.0840164	152.831175	13.83668445
35	cART	350-500	y4	113	416.9100295	206.660779	19.44101075
36	cART	350-500	y5	145	406.1965517	177.6663641	14.75438846
37	cART	350-500	y6	139	436.3621103	197.2908608	16.7339924
38	cART	350-500	y7	149	443.2936242	190.9178673	15.64060111
39	cART	350-500	y8	145	463.366092	190.0451965	15.78239454
40	cART	350-500	y9	143	443.1515152	165.2487868	13.81879776
41	cART	350-500	y10	137	462.8527981	172.3887051	14.72816103
42	cART	350-500	y11	136	471.3038958	190.4273928	16.32901422
43	cART	350-500	y12	137	524.4890511	201.558724	17.22032393
44	cART	350-500	y13	130	505.1576923	200.2821216	17.56590408
45	cART	350-500	y14	132	467.1319444	175.0484148	15.23600886
46	cART	500+	cd4b	125	673.72	157.3403748	14.07295095
47	cART	500+	y1	39	529.2948718	233.897293	37.4535417
48	cART	500+	y2	103	478.6407767	205.4429294	20.24289334
49	cART	500+	y3	88	472.5606061	197.3540267	21.03800994
50	cART	500+	y4	79	580.892827	215.2222759	24.21439787

51	cART	500+	y5	110	514.8582424	209.3243837	19.95829689
52	cART	500+	y6	110	513.7609091	226.9864018	21.64230424
53	cART	500+	y7	113	494.6325959	226.5908466	21.3158738
54	cART	500+	y8	117	539.0837607	216.4423262	20.01010014
55	cART	500+	y9	117	498.2435287	203.2733079	18.79262397
56	cART	500+	y10	115	504.4630435	238.8017345	22.26837657
57	cART	500+	y11	113	534.5423451	224.1688397	21.08803055
58	cART	500+	y12	103	581.0307443	237.8540942	23.43646029
59	cART	500+	y13	95	552.6666667	213.4914626	21.9037619
60	cART	500+	y14	103	531.7200647	215.0964713	21.19408508
61	TCM+cART	200-	cd4b	50	125	59.12102772	8.360975922
62	TCM+cART	200-	y1	27	236.5679012	183.6269459	35.33902221
63	TCM+cART	200-	y2	44	260.4386364	210.0870597	31.67181593
64	TCM+cART	200-	y3	37	377.6563063	234.8521694	38.60945882
65	TCM+cART	200-	y4	37	327.6891892	176.532663	29.02179103
66	TCM+cART	200-	y5	42	373.3218254	156.8161317	24.19725445
67	TCM+cART	200-	y6	43	349.524031	162.2279386	24.73952871
68	TCM+cART	200-	y7	41	324.6247967	121.3485098	18.95145328
69	TCM+cART	200-	y8	42	332.5285714	124.7446552	19.24851819
70	TCM+cART	200-	y9	41	395.3349594	134.0219591	20.93071353
71	TCM+cART	200-	y10	39	421.6235043	168.3428634	26.95643192
72	TCM+cART	200-	y11	38	430.7065614	179.1930829	29.06895656
73	TCM+cART	200-	y12	37	426.2274775	165.5985457	27.22423321
74	TCM+cART	200-	y13	37	377.9238288	170.0607753	27.95781924
75	TCM+cART	200-	y14	37	412.3873874	141.9707415	23.33984613
76	TCM+cART	200-350	cd4b	94	275.8599291	42.33265108	4.366279573
77	TCM+cART	200-350	y1	51	325.3366013	174.7548553	24.47057434
78	TCM+cART	200-350	y2	82	297.5792683	176.5372594	19.49527897
79	TCM+cART	200-350	y3	80	337.3270833	218.1991816	24.39541014
80	TCM+cART	200-350	y4	73	340.0426941	211.7216133	24.78014051
81	TCM+cART	200-350	y5	80	363.48875	189.9764546	21.24001333
82	TCM+cART	200-350	y6	79	368.3911694	190.6795883	21.45312977
83	TCM+cART	200-350	y7	79	363.6402954	203.1022232	22.8507854
84	TCM+cART	200-350	y8	76	392.3217105	196.6722236	22.55985125
85	TCM+cART	200-350	y9	74	441.8716216	210.6146411	24.48345472
86	TCM+cART	200-350	y10	75	490.6955556	191.4413146	22.1057389
87	TCM+cART	200-350	y11	73	455.6609406	217.3860168	25.44310879
88	TCM+cART	200-350	y12	69	489.2995169	231.8949161	27.9168673
89	TCM+cART	200-350	y13	68	461.3406863	218.9370436	26.55001635
90	TCM+cART	200-350	y14	69	458.0241546	203.1634562	24.45800599
91	TCM+cART	350-500	cd4b	84	421.0019841	42.60546795	4.648637665
92	TCM+cART	350-500	y1	42	416.4285714	188.3581362	29.0642914
93	TCM+cART	350-500	y2	70	324.0452381	158.1429008	18.90169194
94	TCM+cART	350-500	y3	67	411.8432836	196.0744548	23.95430719
95	TCM+cART	350-500	y4	72	417.6993056	181.4221122	21.38080096
96	TCM+cART	350-500	y5	77	446.671645	179.0349957	20.40293132
97	TCM+cART	350-500	y6	78	425.1194444	178.2758423	20.1857642
98	TCM+cART	350-500	y7	77	402.0948052	161.9212852	18.452643
99	TCM+cART	350-500	y8	78	445.0271368	198.8067073	22.5104269
100	TCM+cART	350-500	y9	72	431.8138889	206.9482185	24.38908145
101	TCM+cART	350-500	y10	70	498.0561905	200.4122435	23.9538447

102	TCM+cART	350-500	y11	70	460.4595048	189.5965875	22.66112656
103	TCM+cART	350-500	y12	69	487.4299517	229.8532023	27.67107384
104	TCM+cART	350-500	y13	67	441.3233831	195.7254289	23.91166689
105	TCM+cART	350-500	y14	69	450.8635266	209.3711015	25.20531867
106	TCM+cART	500+	cd4b	79	677.6265823	143.0942282	16.09935848
107	TCM+cART	500+	y1	40	511.3625	283.1624067	44.77190764
108	TCM+cART	500+	y2	66	530.1578283	215.058253	26.471841
109	TCM+cART	500+	y3	65	531.9435897	250.3998439	31.05827818
110	TCM+cART	500+	y4	68	543.6958333	238.6543112	28.94108627
111	TCM+cART	500+	y5	72	499.6451389	194.8735062	22.96606295
112	TCM+cART	500+	y6	69	508.5074879	215.058271	25.88997342
113	TCM+cART	500+	y7	69	462.2289855	192.8152747	23.21223133
114	TCM+cART	500+	y8	67	499.6037313	200.645591	24.51276037
115	TCM+cART	500+	y9	69	493.4352657	187.5373621	22.57684532
116	TCM+cART	500+	y10	66	569.084596	227.906839	28.05339261
117	TCM+cART	500+	y11	62	531.5775323	193.3688839	24.55787281
118	TCM+cART	500+	y12	61	566.3087432	206.7576694	26.4726069
119	TCM+cART	500+	y13	59	530.8248588	191.1824804	24.88983893
120	TCM+cART	500+	y14	58	587.3850575	203.4137512	26.70953407
