# Supplementary material for: Effect of Traditional Chinese Medicine Therapy on the Trend in CD4+ T-Cell Counts among Patients with HIV/AIDS Treated with Antiretroviral Therapy: A Retrospective Cohort Study
Source: Evid Based Complement Alternat Med. 2021 Jul 15;2021:5576612. doi: 10.1155/2021/5576612 (PMC8302365; doi:10.1155/2021/5576612)
Supplement: Supplementary Materials — S1: the annual CD4+ T-cell count stratified on baseline CD4+ T-cell count. S2: the annual CD4+ T-cell count stratified on gender. S3: the annual CD4+ T-cell count stratified on age. [file 5576612.f1.zip › 5576612.f1/S2. The annual CD4+T cell count stratified on gender.pdf]

group	cd4cat	sex	time	n	mean	sd	se	
1	cART	200-	female	cd4b	96	121.3489583	60.61214573	6.186201218
2	cART	200-	female	y1	39	175.2435897	166.5355925	26.66703697
3	cART	200-	female	y2	54	195.3672839	143.1933475	19.48614645
4	cART	200-	female	y3	56	214.1964286	173.1514871	23.13834074
5	cART	200-	female	y4	44	224.5416667	171.0991225	25.79416324
6	cART	200-	female	y5	64	344.0643229	198.9685962	24.87107453
7	cART	200-	female	y6	63	395.7513228	185.1597504	23.32793583
8	cART	200-	female	y7	62	394.5403226	219.784554	27.91266627
9	cART	200-	female	y8	62	450.5967742	221.1938717	28.0916498
10	cART	200-	female	y9	61	424.418306	214.3562578	27.44550644
11	cART	200-	female	y10	59	488.4661017	221.9650049	28.89738226
12	cART	200-	female	y11	55	463.5181818	223.1510166	30.08967694
13	cART	200-	female	y12	55	518.2606061	213.9380866	28.84740571
14	cART	200-	female	y13	55	509.9090909	203.3295102	27.41694557
15	cART	200-	female	y14	54	514.4567901	201.5896968	27.43288303
16	cART	200-	male	cd4b	110	119.9590909	56.33932792	5.371744148
17	cART	200-	male	y1	39	167.1410256	140.2847175	22.46353282
18	cART	200-	male	y2	64	184.6979167	120.2880504	15.03600629
19	cART	200-	male	y3	70	199.497619	140.064143	16.74086709
20	cART	200-	male	y4	57	219.9327485	124.3346413	16.46852555
21	cART	200-	male	y5	86	235.2606589	140.3492349	15.13424722
22	cART	200-	male	y6	80	271.315625	149.8900398	16.75821591
23	cART	200-	male	y7	72	301.5775463	167.4828497	19.73804313
24	cART	200-	male	y8	70	344.6359524	182.4027782	21.80130189
25	cART	200-	male	y9	71	343.8037559	185.8909782	22.06120033
26	cART	200-	male	y10	71	363.2699531	178.7662155	21.21564657
27	cART	200-	male	y11	66	335.6304545	171.7759353	21.14415599
28	cART	200-	male	y12	64	370.9739583	186.0251951	23.25314939
29	cART	200-	male	y13	60	366.4611111	184.0789879	23.76449515
30	cART	200-	male	y14	60	358.2138889	182.51517	23.56260713
31	cART	200-350	female	cd4b	121	270.6239669	44.45542154	4.041401958
32	cART	200-350	female	y1	41	263.9878049	117.9654339	18.42310559
33	cART	200-350	female	y2	73	305.9200913	143.2723064	16.76875511
34	cART	200-350	female	y3	85	302.1117647	135.8465263	14.73462457
35	cART	200-350	female	y4	73	339.2625571	160.5554929	18.79159908
36	cART	200-350	female	y5	108	361.4577381	176.4172016	16.97575314
37	cART	200-350	female	y6	106	403.8455975	182.2246903	17.69922654
38	cART	200-350	female	y7	101	408.1980198	180.9629074	18.00648229
39	cART	200-350	female	y8	101	405.6427393	179.7645541	17.88724168
40	cART	200-350	female	y9	100	410.5675	183.5940157	18.35940157
41	cART	200-350	female	y10	100	461.8383333	196.8658046	19.68658046
42	cART	200-350	female	y11	94	470.6683333	186.4441719	19.23024801
43	cART	200-350	female	y12	93	505.0053763	219.0295218	22.71230339
44	cART	200-350	female	y13	85	491.5941176	177.8388643	19.28933313
45	cART	200-350	female	y14	89	471.6685393	195.4915643	20.72206437
46	cART	200-350	male	cd4b	102	273.9313725	46.8989349	4.643686516
47	cART	200-350	male	y1	29	247.0689655	122.0883553	22.67123843
48	cART	200-350	male	y2	71	254.5211268	132.1395935	15.68208459
49	cART	200-350	male	y3	64	283.3020833	138.7658912	17.3457364
50	cART	200-350	male	y4	58	287.316092	146.6386692	19.25460057

51	cART	200-350	male	y5	84	333.1928571	161.0529471	17.57231717
52	cART	200-350	male	y6	83	350.4307229	185.3601516	20.34591987
53	cART	200-350	male	y7	77	340.1612554	171.6361572	19.55975542
54	cART	200-350	male	y8	78	399.840812	180.862693	20.47866736
55	cART	200-350	male	y9	77	386.2692641	183.4896354	20.91058463
56	cART	200-350	male	y10	76	430.6118421	209.9389283	24.08164665
57	cART	200-350	male	y11	69	448.6420411	213.3613863	25.6856925
58	cART	200-350	male	y12	69	463.0845411	220.0949098	26.49631347
59	cART	200-350	male	y13	67	429.8507463	206.6180868	25.24241686
60	cART	200-350	male	y14	67	474.6728856	226.6725324	27.69245733
61	TCM+cART	200-	female	cd4b	23	145.5434783	55.58652409	11.5905915
62	TCM+cART	200-	female	y1	13	264.5384615	215.1409869	59.66937382
63	TCM+cART	200-	female	y2	20	272.525	188.5426432	42.15941668
64	TCM+cART	200-	female	y3	19	456.8245614	257.5498003	59.08597643
65	TCM+cART	200-	female	y4	16	333.3333333	193.758412	48.439603
66	TCM+cART	200-	female	y5	19	404.2184211	175.001492	40.14809573
67	TCM+cART	200-	female	y6	20	354.0041667	164.7273689	36.83415947
68	TCM+cART	200-	female	y7	18	348.5648148	128.5152027	30.29132377
69	TCM+cART	200-	female	y8	19	360.5	134.6702362	30.89547108
70	TCM+cART	200-	female	y9	18	419.9768519	168.8144764	39.78995368
71	TCM+cART	200-	female	y10	18	487.0546296	189.9828633	44.77939032
72	TCM+cART	200-	female	y11	17	511.7993726	200.2578393	48.56966022
73	TCM+cART	200-	female	y12	18	457.3425926	150.1897	35.40005178
74	TCM+cART	200-	female	y13	18	413.6582407	199.9416744	47.12670461
75	TCM+cART	200-	female	y14	18	412.8055556	111.0622691	26.17762787
76	TCM+cART	200-	male	cd4b	27	107.5	57.28370691	11.02425453
77	TCM+cART	200-	male	y1	14	210.5952381	152.3275361	40.71124649
78	TCM+cART	200-	male	y2	24	250.3666667	230.0376258	46.95623373
79	TCM+cART	200-	male	y3	18	294.0898148	179.351808	42.27362654
80	TCM+cART	200-	male	y4	21	323.3888889	167.0124607	36.44510682
81	TCM+cART	200-	male	y5	23	347.7985507	138.8038854	28.94261084
82	TCM+cART	200-	male	y6	23	345.6282609	163.6285221	34.11890541
83	TCM+cART	200-	male	y7	23	305.8891304	114.7831828	23.93394811
84	TCM+cART	200-	male	y8	23	309.4217391	113.7011987	23.70833883
85	TCM+cART	200-	male	y9	23	376.05	98.74667326	20.59010471
86	TCM+cART	200-	male	y10	21	365.5396825	126.5285283	27.61078851
87	TCM+cART	200-	male	y11	21	365.06	131.1741363	28.62454327
88	TCM+cART	200-	male	y12	19	396.75	177.9230982	40.81835814
89	TCM+cART	200-	male	y13	19	344.0701754	132.6608425	30.43448453
90	TCM+cART	200-	male	y14	19	411.9912281	169.2970258	38.83940141
91	TCM+cART	200-350	female	cd4b	40	280.7125	36.93161825	5.839401568
92	TCM+cART	200-350	female	y1	19	367.2017544	176.5110826	40.49441955
93	TCM+cART	200-350	female	y2	35	283.5785714	140.4041129	23.73262668
94	TCM+cART	200-350	female	y3	32	420.0572917	227.0581518	40.13858972
95	TCM+cART	200-350	female	y4	29	369.2045977	203.8986782	37.86303401
96	TCM+cART	200-350	female	y5	36	426.912037	216.3622283	36.06037139
97	TCM+cART	200-350	female	y6	35	429.2519048	205.4836305	34.73307292
98	TCM+cART	200-350	female	y7	35	408.9785714	217.5724208	36.77645144
99	TCM+cART	200-350	female	y8	34	427.7877451	221.9357772	38.06167177
100	TCM+cART	200-350	female	y9	32	495.3458333	233.9593577	41.35856209
101	TCM+cART	200-350	female	y10	33	568.0075758	175.6459746	30.57603954

102	TCM+cART	200-350	female	y11	32	498.48775	244.0004674	43.13359629
103	TCM+cART	200-350	female	y12	31	549.5860215	234.1844037	42.06076056
104	TCM+cART	200-350	female	y13	30	507.6388889	230.8053121	42.13909195
105	TCM+cART	200-350	female	y14	31	499.1827957	212.9397992	38.24511694
106	TCM+cART	200-350	male	cd4b	53	270.7987421	45.0791725	6.192100557
107	TCM+cART	200-350	male	y1	31	299.9784946	174.4352514	31.32949601
108	TCM+cART	200-350	male	y2	46	311.3097826	201.0288757	29.64009067
109	TCM+cART	200-350	male	y3	47	285.1241135	196.6222391	28.68030124
110	TCM+cART	200-350	male	y4	43	324.074031	218.3521958	33.2983977
111	TCM+cART	200-350	male	y5	43	311.8782946	150.3416906	22.92689287
112	TCM+cART	200-350	male	y6	43	318.7810631	166.5403269	25.39716176
113	TCM+cART	200-350	male	y7	43	322.3565891	184.3486265	28.11290199
114	TCM+cART	200-350	male	y8	41	362.1138211	172.7945691	26.98597789
115	TCM+cART	200-350	male	y9	41	401.1630081	185.7487059	29.00907416
116	TCM+cART	200-350	male	y10	41	435.6077236	181.4867211	28.34346397
117	TCM+cART	200-350	male	y11	40	423.8410167	192.695296	30.4678015
118	TCM+cART	200-350	male	y12	38	440.1184211	220.9845969	35.84843535
119	TCM+cART	200-350	male	y13	38	424.7894737	204.7810106	33.21986655
120	TCM+cART	200-350	male	y14	38	424.4473684	191.0900103	30.99889304
