# Supplementary material for: Effect of Traditional Chinese Medicine Therapy on the Trend in CD4+ T-Cell Counts among Patients with HIV/AIDS Treated with Antiretroviral Therapy: A Retrospective Cohort Study
Source: Evid Based Complement Alternat Med. 2021 Jul 15;2021:5576612. doi: 10.1155/2021/5576612 (PMC8302365; doi:10.1155/2021/5576612)
Supplement: Supplementary Materials — S1: the annual CD4+ T-cell count stratified on baseline CD4+ T-cell count. S2: the annual CD4+ T-cell count stratified on gender. S3: the annual CD4+ T-cell count stratified on age. [file 5576612.f1.zip › 5576612.f1/S3. The annual CD4+T cell count stratified on age.pdf]

group	cd4cat	agecat	time	n	mean	sd	se	
1	cART	200-	<40years	cd4b	111	121.7657658	58.66041796	5.567800474
2	cART	200-	<40years	y1	50	174.83	161.7757837	22.87855073
3	cART	200-	<40years	y2	60	191.075	146.3025218	18.88757435
4	cART	200-	<40years	y3	67	188.4502488	162.6072037	19.86563173
5	cART	200-	<40years	y4	54	222.8364198	153.9984736	20.95653786
6	cART	200-	<40years	y5	80	265.3397917	183.4907035	20.51488431
7	cART	200-	<40years	y6	76	320.5910088	176.5524763	20.25195796
8	cART	200-	<40years	y7	69	324.3985507	191.161904	23.01318889
9	cART	200-	<40years	y8	71	378.2664319	196.587331	23.33062386
10	cART	200-	<40years	y9	72	392.8944444	208.0115631	24.51439781
11	cART	200-	<40years	y10	71	415.2370892	202.4767646	24.02957105
12	cART	200-	<40years	y11	66	371.8400505	193.4925449	23.81728584
13	cART	200-	<40years	y12	66	426.6742424	200.4965789	24.67942283
14	cART	200-	<40years	y13	63	434.2698413	215.983251	27.21133188
15	cART	200-	<40years	y14	63	420.2751323	196.0483449	24.69976979
16	cART	200-	>=40years	cd4b	95	119.2526316	58.00347906	5.951031386
17	cART	200-	>=40years	y1	28	164.6964286	138.6130598	26.19540606
18	cART	200-	>=40years	y2	58	188.0344828	113.8243053	14.9458635
19	cART	200-	>=40years	y3	59	225.9943503	145.0330157	18.88169037
20	cART	200-	>=40years	y4	47	220.9113475	137.3698719	20.0374552
21	cART	200-	>=40years	y5	70	300.3621429	165.816041	19.81880761
22	cART	200-	>=40years	y6	67	332.4278607	178.52307	21.81006426
23	cART	200-	>=40years	y7	65	366.024359	204.7557232	25.39682178
24	cART	200-	>=40years	y8	61	413.1901639	220.003346	28.16854199
25	cART	200-	>=40years	y9	60	366.8530556	197.2155761	25.4604214
26	cART	200-	>=40years	y10	59	425.9293785	216.8024005	28.22526841
27	cART	200-	>=40years	y11	55	420.0666667	219.1854745	29.55496335
28	cART	200-	>=40years	y12	53	454.4559748	226.0770285	31.05406813
29	cART	200-	>=40years	y13	52	436.0320513	194.5282491	26.97621449
30	cART	200-	>=40years	y14	51	446.9836601	219.5435752	30.74224959
31	cART	200-350	<40years	cd4b	123	270.5772358	46.52022443	4.194587376
32	cART	200-350	<40years	y1	40	246.4	111.1604984	17.57601804
33	cART	200-350	<40years	y2	79	275.021097	132.7418058	14.93461997
34	cART	200-350	<40years	y3	81	283.9670782	131.8277928	14.64753253
35	cART	200-350	<40years	y4	73	297.1666667	151.3264219	17.71141802
36	cART	200-350	<40years	y5	106	347.8214061	168.0413125	16.32161512
37	cART	200-350	<40years	y6	103	372.7014563	176.007023	17.3424873
38	cART	200-350	<40years	y7	101	367.0016502	175.7205669	17.48484992
39	cART	200-350	<40years	y8	104	406.8717949	187.8153744	18.41681268
40	cART	200-350	<40years	y9	102	398.8707516	182.4641277	18.06664077
41	cART	200-350	<40years	y10	103	481.6375405	205.6132485	20.25967537
42	cART	200-350	<40years	y11	97	485.7770447	205.5479087	20.87022809
43	cART	200-350	<40years	y12	98	501.1972789	228.3592949	23.06777228
44	cART	200-350	<40years	y13	89	464.6853933	195.727893	20.74711517
45	cART	200-350	<40years	y14	94	492.8510638	229.5324991	23.67446963
46	cART	200-350	>=40years	cd4b	100	274.055	44.40617705	4.440617705
47	cART	200-350	>=40years	y1	30	271.0833333	129.5213392	23.64725305
48	cART	200-350	>=40years	y2	65	287.3307692	148.7182987	18.44623471
49	cART	200-350	>=40years	y3	68	306.0220588	142.8879343	17.32770723
50	cART	200-350	>=40years	y4	58	340.2988506	160.0435758	21.01475104

51	cART	200-350	>=40years	y5	86	350.6577519	173.3887487	18.69698962
52	cART	200-350	>=40years	y6	86	389.5945736	195.9482609	21.12964438
53	cART	200-350	>=40years	y7	77	394.1980519	184.8113305	21.06120576
54	cART	200-350	>=40years	y8	75	397.9044444	169.0591128	19.52126485
55	cART	200-350	>=40years	y9	75	401.5288889	185.9432251	21.47087422
56	cART	200-350	>=40years	y10	73	401.3926941	189.9273403	22.22931379
57	cART	200-350	>=40years	y11	66	425.4356187	181.8150119	22.3798809
58	cART	200-350	>=40years	y12	64	465.640625	205.8645045	25.73306306
59	cART	200-350	>=40years	y13	63	463.9444444	190.3237221	23.9785351
60	cART	200-350	>=40years	y14	62	442.7997312	169.9197332	21.57982769
61	`CM+cAR′	200-	<40years	cd4b	26	120.3076923	64.41569326	12.6329568
62	`CM+cAR′	200-	<40years	y1	11	193.6666667	186.8481261	56.33682973
63	`CM+cAR′	200-	<40years	y2	24	298.7208333	257.6218428	52.58683846
64	`CM+cAR′	200-	<40years	y3	18	405.8166667	286.2315207	67.46541642
65	`CM+cAR′	200-	<40years	y4	20	349.2083333	169.4013615	37.87929597
66	`CM+cAR′	200-	<40years	y5	21	387.402381	154.4551674	33.70488076
67	`CM+cAR′	200-	<40years	y6	22	360.8757576	149.5265144	31.87915999
68	`CM+cAR′	200-	<40years	y7	22	304.6795455	141.9367573	30.26101833
69	`CM+cAR′	200-	<40years	y8	22	326.969697	142.1644861	30.30957027
70	`CM+cAR′	200-	<40years	y9	21	365.2769841	114.9434493	25.08271701
71	`CM+cAR′	200-	<40years	y10	22	356.2575758	126.0702986	26.87827796
72	`CM+cAR′	200-	<40years	y11	21	397.8499683	191.8486208	41.86480128
73	`CM+cAR′	200-	<40years	y12	20	419.1791667	177.7124506	39.73771201
74	`CM+cAR′	200-	<40years	y13	21	365.3499206	159.2039355	34.74114693
75	`CM+cAR′	200-	<40years	y14	21	398.0396825	162.7369034	35.51210373
76	`CM+cAR′	200-	>=40years	cd4b	24	130.0833333	53.70706914	10.96290958
77	`CM+cAR′	200-	>=40years	y1	16	266.0625	181.3658251	45.34145627
78	`CM+cAR′	200-	>=40years	y2	20	214.5	124.3944279	27.81543967
79	`CM+cAR′	200-	>=40years	y3	19	350.9780702	177.1719709	40.64603773
80	`CM+cAR′	200-	>=40years	y4	17	302.372549	186.4971843	45.23221116
81	`CM+cAR′	200-	>=40years	y5	21	359.2412698	161.6768823	35.28078815
82	`CM+cAR′	200-	>=40years	y6	21	337.631746	177.4859585	38.73061141
83	`CM+cAR′	200-	>=40years	y7	19	347.7192982	90.38985868	20.73685576
84	`CM+cAR′	200-	>=40years	y8	20	338.6433333	105.6700913	23.62855074
85	`CM+cAR′	200-	>=40years	y9	20	426.8958333	147.8513826	33.06057421
86	`CM+cAR′	200-	>=40years	y10	17	506.2147059	181.5695645	44.03708782
87	`CM+cAR′	200-	>=40years	y11	17	471.2941176	158.3671513	38.40967603
88	`CM+cAR′	200-	>=40years	y12	17	434.5196078	155.1226428	37.62276713
89	`CM+cAR′	200-	>=40years	y13	16	394.4270833	187.358529	46.83963226
90	`CM+cAR′	200-	>=40years	y14	16	431.21875	111.3379194	27.83447984
91	`CM+cAR′	200-350	<40years	cd4b	46	267.9057971	41.40304235	6.104545555
92	`CM+cAR′	200-350	<40years	y1	21	299.952381	171.6454125	37.45609978
93	`CM+cAR′	200-350	<40years	y2	39	301.3504274	211.5175735	33.86991855
94	`CM+cAR′	200-350	<40years	y3	39	292.3247863	207.5451686	33.23382468
95	`CM+cAR′	200-350	<40years	y4	38	318.1447368	232.7414724	37.75565241
96	`CM+cAR′	200-350	<40years	y5	39	304.3615385	157.0736978	25.15192124
97	`CM+cAR′	200-350	<40years	y6	39	350.4257021	168.1908402	26.93208872
98	`CM+cAR′	200-350	<40years	y7	40	332.5470833	166.6611443	26.35144068
99	`CM+cAR′	200-350	<40years	y8	40	366.5445833	165.4511101	26.16011746
100	`CM+cAR′	200-350	<40years	y9	40	414.57125	187.6685765	29.67300734
101	`CM+cAR′	200-350	<40years	y10	40	466.1291667	175.7065741	27.78164871

102	`CM+cAR′200-350	<40years	y11	40	429.3276833	193.1492857	30.53958356
103	`CM+cAR′200-350	<40years	y12	38	483.3201754	196.2672245	31.83874807
104	`CM+cAR′200-350	<40years	y13	39	437.517094	206.0003186	32.98645071
105	`CM+cAR′200-350	<40years	y14	39	434.9615385	171.5793316	27.47468161
106	`CM+cAR′200-350	>=40years	cd4b	47	282.0673759	41.52089951	6.056445654
107	`CM+cAR′200-350	>=40years	y1	29	344.0402299	180.6690314	33.54939689
108	`CM+cAR′200-350	>=40years	y2	42	297.4484127	140.0078116	21.60367435
109	`CM+cAR′200-350	>=40years	y3	40	386.05	221.4695068	35.01740369
110	`CM+cAR′200-350	>=40years	y4	34	369.1946078	186.7445455	32.02642533
111	`CM+cAR′200-350	>=40years	y5	40	422.7375	204.6131715	32.35218307
112	`CM+cAR′200-350	>=40years	y6	39	386.2769231	213.7359201	34.22513828
113	`CM+cAR′200-350	>=40years	y7	38	391.4131579	234.2796304	38.00517458
114	`CM+cAR′200-350	>=40years	y8	35	420.847619	228.6328806	38.64601035
115	`CM+cAR′200-350	>=40years	y9	33	476.2393939	236.7783641	41.21782261
116	`CM+cAR′200-350	>=40years	y10	34	528.2058824	202.2895061	34.69236409
117	`CM+cAR′200-350	>=40years	y11	32	491.6294167	245.5308329	43.40412923
118	`CM+cAR′200-350	>=40years	y12	31	496.6290323	272.544963	48.9505204
119	`CM+cAR′200-350	>=40years	y13	29	493.3793103	235.0580513	43.64918433
120	`CM+cAR′200-350	>=40years	y14	30	488.0055556	237.8290279	43.42144114
